# Extreme mutational selectivity of axitinib limits its potential use as a targeted therapeutic for BCR-ABL1-positive leukemia

**DOI:** 10.1038/leu.2015.318

**Published:** 2015-12-08

**Authors:** M S Zabriskie, C A Eide, D Yan, N A Vellore, A D Pomicter, S L Savage, B J Druker, M W Deininger, T O'Hare

**Affiliations:** 1 Huntsman Cancer Institute, University of Utah, Salt Lake City, UT, USA; 2 Knight Cancer Institute, Oregon Health & Science University, Portland, OR, USA; 3 Howard Hughes Medical Institute, Portland, OR, USA; 4 Division of Hematology and Hematologic Malignancies, University of Utah, Salt Lake City, UT, USA

Clinical use of the BCR-ABL1 selective tyrosine kinase inhibitor (TKI) imatinib has markedly improved the prognosis of chronic myeloid leukemia (CML). Next-generation TKIs, including nilotinib, dasatinib, bosutinib and ponatinib, effectively control resistance due to BCR-ABL1 point mutations. Ponatinib, the only US Food and Drug Administration (FDA)-approved TKI with activity against the clinically prevalent BCR-ABL1^T315I^ mutant,^1^ has been linked to severe vascular occlusive events^2, 3, 4, 5, 6^ at a dose of 45 mg once daily, and lower doses are being explored (clinicaltrials.gov identifier NCT02467270). Emergence of BCR-ABL1 compound mutations can confer high-level resistance to all available TKIs, including ponatinib, indicating a need for new therapeutic options.^7^

Axitinib, an FDA-approved, ATP-competitive inhibitor of vascular endothelial growth factor receptors (VEGFR) 1, 2 and 3, is used to treat metastatic renal cell carcinoma after prior treatment failure with sorafenib or systemic therapies.^8, 9, 10^ Recent interest in repositioning FDA-approved drugs led to the discovery that axitinib has activity against BCR-ABL1^T315I^.^11^ In contrast to all FDA-approved TKIs currently used in CML, Pemovska *et al.*^11^ reported that axitinib is inactive against native BCR-ABL1. In consideration of axitinib's extreme selectivity for BCR-ABL1^T315I^, we explored its efficacy against other BCR-ABL1 point mutations, T315I-inclusive compound mutations and secondary mutations of T315.

Upon profiling axitinib against a panel of Ba/F3 cells expressing native or single-mutant BCR-ABL1 (Figure 1a, left; and 1b; Supplementary Table S1), we found that only three mutants exhibited a half maximal inhibitory concentration (IC_50_)<500 nm, with V299L (IC_50_: 236 nm) being the only substitution away from position 315. In the recent report, IC_50_ values of T315V, T315I and T315A were 45, 98 and 389 nm, respectively.^11^ We extended this survey to include two additional clinically observed mutants, T315L and T315M. In the present study, both T315I and T315L (IC_50_: 146  and 201 nm, respectively) may represent potential axitinib targets depending on achievable steady-state levels (Figure 1a), whereas the T315M mutant (IC_50_: 736 nm) is highly resistant to axitinib. Thus, only certain substitutions are tolerated at position 315, further indicating this residue represents an important determinant of axitinib binding (Supplementary Figures 1a–c). Immunoblot analysis confirmed inhibition of BCR-ABL1 phosphorylation by axitinib for the T315I mutant, but not for native BCR-ABL1 or the T315M mutant (Figure 1c).

Analysis of a panel of clinically observed T315I-inclusive compound mutants (M244V/T315I, G250E/T315I, Q252H/T315I, Y253H/T315I, E255V/T315I, F311I/T315I, T315I/M351T, T315I/F359V, T315I/H396R and T315I/E453K) revealed several instances in which axitinib is substantially more potent against the compound mutant than either component mutant (Figure 1a, center; and 1b; Supplementary Figure S2), including T315I/H396R (IC_50_: 79 vs 146 and 565 nm for T315I and H396R, respectively) and M244V/T315I (IC_50_: 83 vs 567 and 146 for M244V and T315I, respectively). Axitinib may find utility in these settings, depending on achievable plasma concentrations (Figure 1a).^8^ Most compound mutants involving the P-loop (for example, G250E/T315I and Y253H/T315I) were significantly less sensitive compared with T315I alone (Supplementary Figure 1b and d). Overall, axitinib was much more effective against T315I-inclusive compound mutants than the corresponding non-T315I single mutants, in line with axitinib more potently inhibiting BCR-ABL1^T315I^ than native BCR-ABL1.

Among a panel of non-T315I compound mutants (G250E/V299L, Y253H/E255V, Y253H/F317L, E255V/V299L, V299L/F317L, V299L/M351T, V299L/F359V and F317L/F359V; Figure 1a, right; and 1b; Supplementary Figure S2; Supplementary Table S2), only two V299L-inclusive compound mutants were sensitive to axitinib, consistent with V299L as the only single mutant aside from select variants of position 315 that exhibited sensitivity to axitinib. Both of these (V299L/M351T IC_50_: 143 nm; V299L/F359V IC_50_: 147 nm) are also addressed by ponatinib,^7^ but axitinib potentially provides an alternative therapy if ponatinib is not tolerated. All other non-T315I compound mutants tested were outside of the clinically achievable dose range.^8^

To determine whether select secondary acquired mutations on a BCR-ABL1^T315I^ background confer resistance to axitinib, we performed a cell-based accelerated mutagenesis screen of Ba/F3 BCR-ABL1^T315I^ in the presence of increasing concentrations of inhibitor. Axitinib demonstrated concentration-dependent restriction of the outgrowth of resistant clones (Supplementary Figure S2a). Compound mutants recovered included Q252(H;P;R)/T315I and G250E/T315I (recovered at 200 and 400 nm axitinib, respectively; Supplementary Figure S2b), consistent with our cell proliferation panel findings (Q252H/T315I IC_50_: 320 nm; G250E/T315I IC_50_: 762 nm; Figures 1a and b). Of note, two instances of isoleucine-to-threonine reversion at position 315 (axitinib IC_50_: 811 nm) were detected in the presence of 200 nm axitinib, consistent with native BCR-ABL1 conferring resistance to axitinib.

We also explored the potential of a T315I-selective inhibitor such as axitinib to treat BCR-ABL1-positive leukemia characterized by the simultaneous presence of clones expressing either native BCR-ABL1 or BCR-ABL1^T315I^. Ba/F3 cells were mixed at a 7:3 native BCR-ABL1:BCR-ABL1^T315I^ ratio, and then cultured with the indicated TKI(s) for 72 h. Cell counts were monitored and Sanger sequencing analysis was conducted at the beginning and end of the experiment as an approximate measure of the native BCR-ABL1:BCR-ABL1^T315I^ ratio (Figures 2a and b).^12^ Relative to proliferation of untreated cells, axitinib (500 nm) reduced proliferation by 60% at 72 h and skewed the initial 7:3 BCR-ABL1:BCR-ABL1^T315I^ ratio to 9:1. Ponatinib (25 nm) decreased overall cell growth to 20% of untreated control and the remaining cells were exclusively BCR-ABL1^T315I^, in line with greater potency of ponatinib against native BCR-ABL1 compared with BCR-ABL1^T315I^.^7^ Axitinib (250 nm) in combination with dasatinib (5 nm) reduced proliferation to 15% of untreated cells, whereas the BCR-ABL1:BCR-ABL1^T315I^ ratio remained relatively constant. Dasatinib (10 nm) reduced cell proliferation to 85% of untreated cells, but the initial 7:3 ratio favoring native BCR-ABL1 moved to favor T315I, in a ratio of ~1:9 (Figures 2a and b). Overall, neither axitinib nor dasatinib alone was effective in this setting, whereas a \combination of the two was as effective as single-agent ponatinib.

As an extension of our cell line mixing experiments, we further assessed the effects of axitinib (100 nm) alone or in combination with dasatinib (10 nm) in colony assays involving primary CML specimens with varying BCR-ABL1^T315I^ allele burden as estimated by cloning and sequencing (Figure 2c).^7^ Consistent with axitinib's selectivity for the T315I mutant over native BCR-ABL1, the degree of colony inhibition by axitinib alone was tracked with the relative abundance of the T315I mutation (T315I allele burdens of 89%, 39% and 0% reduced colony growth to 56%, 73% and 91% of control, respectively). In contrast, the effect of the combination of axitinib and dasatinib was relatively constant across all three specimens (reduced to 31–51% of control), irrespective of T315I mutant burden (Figure 2c). These results highlight the necessity of including a second TKI to inhibit native BCR-ABL1 in the case of axitinib, introducing the potential for associated toxicity issues.^5, 6^ T315I-positive patients typically have a mix of native BCR-ABL1 and BCR-ABL1^T315I^ at the time of switching to a TKI with T315I activity. For example, among 27 patients with the T315I mutation detected prior to starting ponatinib, the average T315I allele burden was 81.5% and the range was 40–100% (Supplementary Table S3).^4^ Our cell line and *ex vivo* primary CML cell studies suggest that residual native BCR-ABL1-positive clones remain a liability for axitinib (Figure 2).

The IC_50_ values for native BCR-ABL1 and the kinase domain single mutants evaluated in our study exceed the reported steady-state peak plasma level of axitinib dosed at the recommended 5 mg twice daily (78 nm), as well as the maximum allowable dose of 10 mg twice daily (150 nm).^8^ Although direct comparison of pre-clinical IC_50_ values with peak plasma levels of axitinib is not possible, this observation raises an important concern about axitinib's clinical role in CML. In the recent report on axitinib as a BCR-ABL^T315I^ inhibitor, a consideration for preferring axitinib to ponatinib was the possibility of reducing the risk of ponatinib treatment-related thrombotic events. The scientific justification for this assertion is not entirely clear, given that axitinib is a low-nanomolar inhibitor of VEGFR1, 2 and 3.^10^ Prescribing information for axitinib states that the risk of arterial and venous embolic and thrombotic events as well as hypertension must be considered carefully and managed appropriately.^13, 14^ Although the mechanisms responsible for arterial occlusive events associated with ponatinib remain to be established, it is plausible that ponatinib's potent inhibition of VEGFR2 is a contributing factor.^5, 6^

We conclude that the potential clinical utility of axitinib in BCR-ABL1-positive leukemia encompasses mutations at positions 315 or 299 only, with plasma levels of axitinib projected to be insufficient to inhibit native BCR-ABL1 and all other single mutants tested. In fact, containment of T315I (IC_50_: 146 nm) and V299L (IC_50_: 236 nm) requires axitinib concentrations exceeding the clinically attainable plasma levels at the standard 5 mg twice-daily dose. Escalation to a dose of 7–10 mg twice daily is permitted based on individual tolerability.^8, 10^ In principle, axitinib is the only TKI with demonstrated activity against T315L (IC_50_: 201 nm), but this is of uncertain clinical utility due to dosing limitations. For axitinib, T315I is the default sensitive background and native BCR-ABL1 functions as a point mutant with considerable resistance. Although it is possible that useful principles for designing analogs with activity against T315I-inclusive compound mutants can be extracted from the axitinib:BCR-ABL^T315I^ complex, the extreme mutational selectivity of axitinib limits its use as a targeted therapy for BCR-ABL1-positive leukemia.

## Figures and Tables

**Figure 1:**
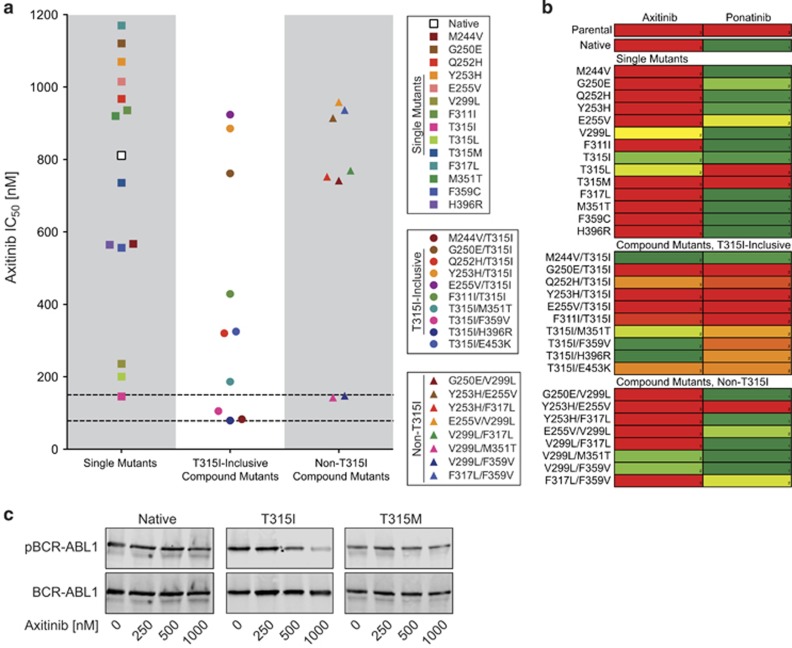
BCR-ABL1 mutant sensitivity profile to axitinib and ponatinib. (**a**) Ba/F3 BCR-ABL1 cells expressing single (left), T315I-inclusive compound (middle) and non-T315I compound (right) mutants were incubated in 96-well plates (2 × 10^3^ cells per well) in twofold escalating concentrations of axitinib (up to 2500 nm) for 72 h. Proliferation was assessed via methanethiosulfonate-based viability assay (CellTiter 96 AQueous One; Promega, Madison, WI, USA). Mean IC_50_ values (Supplementary Table S1) of three independent experiments performed in quadruplicate are plotted. The horizontal dotted lines indicate the reported steady-state plasma C_max_ (78 nm, 5 mg twice daily; 150 nm, 10 mg twice daily) achievable in patients. (**b**) A color gradient from green (sensitive) to yellow (moderately resistant) to red (highly resistant) denotes the IC_50_ sensitivity to each TKI: axitinib (green: <100 nm; yellow: 100–500 nm; red: >500 nm); ponatinib (green: <25 nm; yellow: 25–150 nm; red: >150 nm). Ponatinib results, for reference purposes, are from Zabriskie *et al.*^7^, with exception of T315L, which was determined in the current study. (**c**) Ba/F3 cells expressing native, T315I or T315M were cultured for 6 h in standard medium alone or with escalating concentrations of axitinib. Following axitinib exposure, cells were pelleted and lysed by boiling for 10 min in SDS-polyacrylamide gel electrophoresis loading buffer. Lysates were separated on 4–15% Tris-glycine gels, transferred and immunoblotted with antibodies for the BCR N terminus (Santa Cruz, sc-885, Dallas, TX, USA) and pY412-ABL1 (Santa Cruz, sc-293130).

**Figure 2:**
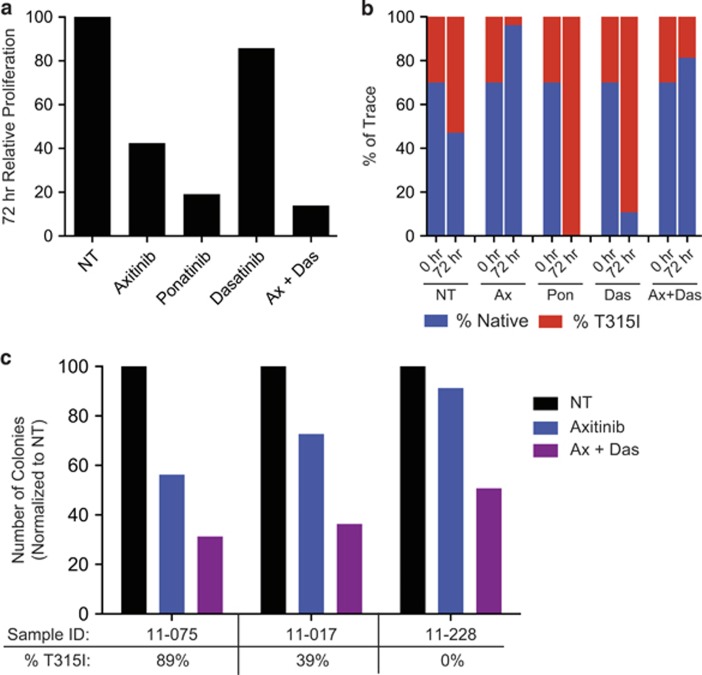
Axitinib effects in a mixed population of BCR-ABL1 native and T315I cells. (**a**) Ba/F3 BCR-ABL1 cells expressing native or T315I were mixed and incubated for 72 h without drug, or with axitinib (500 nm), ponatinib (25 nm), dasatinib (10 nm), or axitinib and dasatinib (250 and 5 nm, respectively). At 0 h and after 72 h, genomic DNA was extracted and the *BCR-ABL1* kinase domain was amplified using two-step PCR to exclude amplification of endogenous *ABL1*. The resulting PCR product was sequenced in both directions using conventional Sanger sequencing and relative native, and T315I expression was evaluated using Mutation Surveyor software (SoftGenetics, State College, PA, USA). (**b**) Using the same cell mixture as in **a**, cells were plated in quadruplicate in 96-well plates (2 × 10^3^ cells per well) without drug, or with axitinib (500 nm), ponatinib (25 nm), dasatinib (10 nm), or axitinib and dasatinib (250 and 5 nm, respectively). After 72 h, relative proliferation was assessed via methanethiosulfonate assay. (**c**) Patient-derived mononuclear cells expressing various levels of BCR-ABL1^T315I^ (89% (left), 39% (middle) or 0% (right)) were plated in methylcellulose semisolid medium with StemSpan CC100 (STEMCELL Technologies, Vancouver, BC, Canada) without drug, with axitinib (100 nm), or with axitinib and dasatinib (100 and 10 nm, respectively). After 2 weeks, colonies were counted.
