# Supplementary figures and images for: Extreme mutational selectivity of axitinib limits its potential use as a targeted therapeutic for BCR-ABL1-positive leukemia
Source: Leukemia. 2015 Dec 8;30(6):1418–21. doi: 10.1038/leu.2015.318 (PMC4873472; doi:10.1038/leu.2015.318)

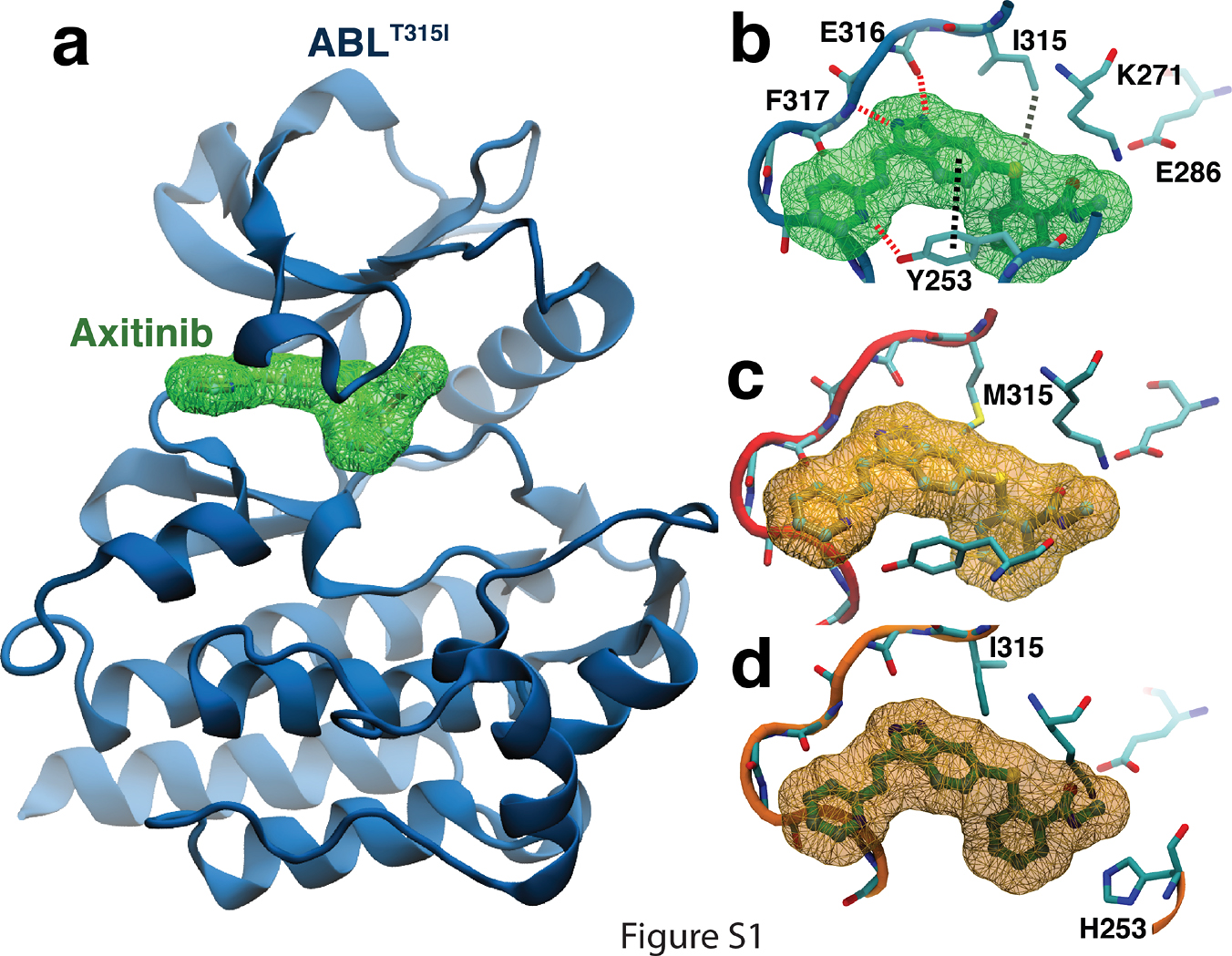

Supplement: Supplementary Figure 1 [file leu2015318x1.tif]

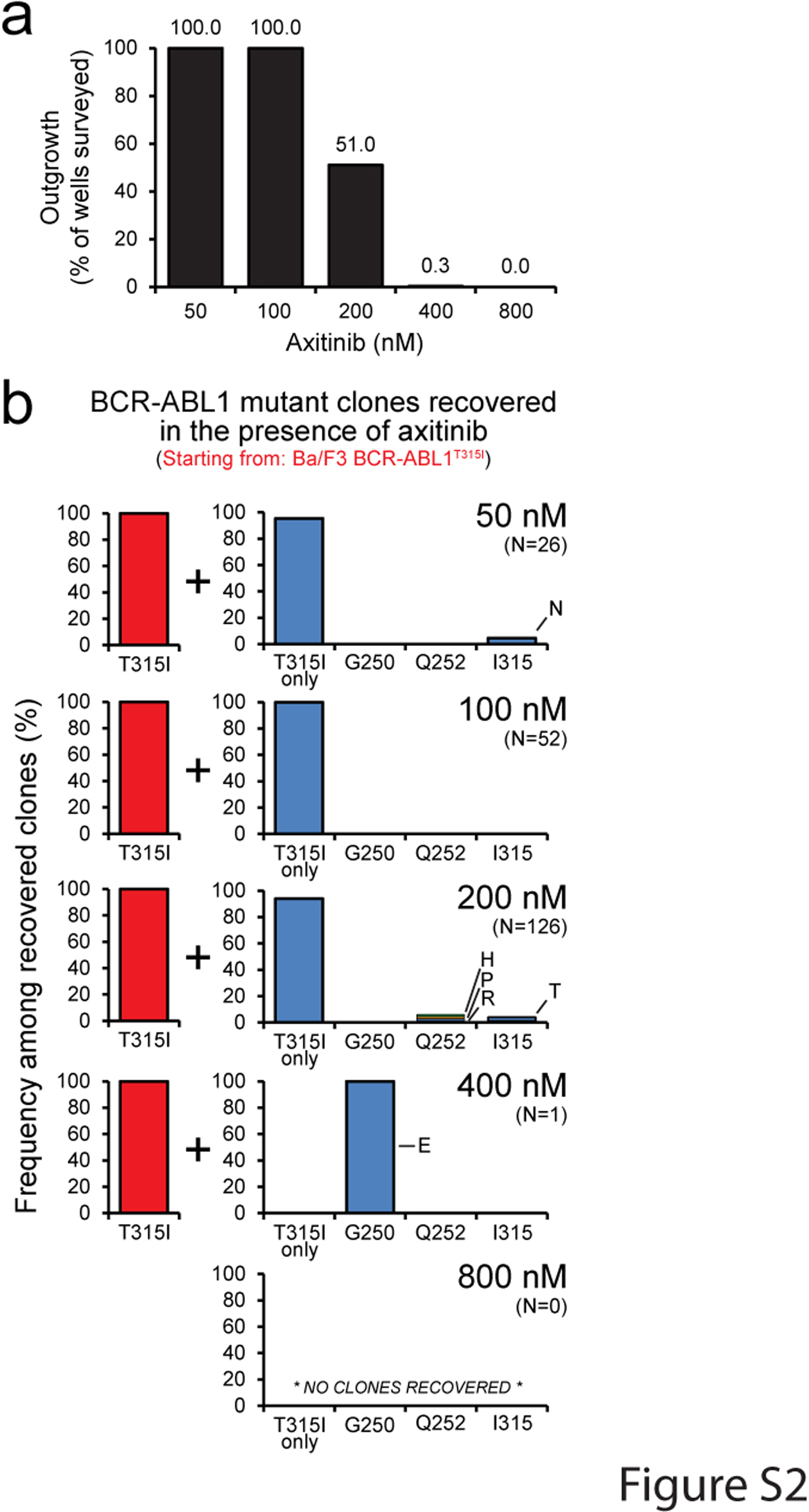

Supplement: Supplementary Figure 2 [file leu2015318x2.tif]

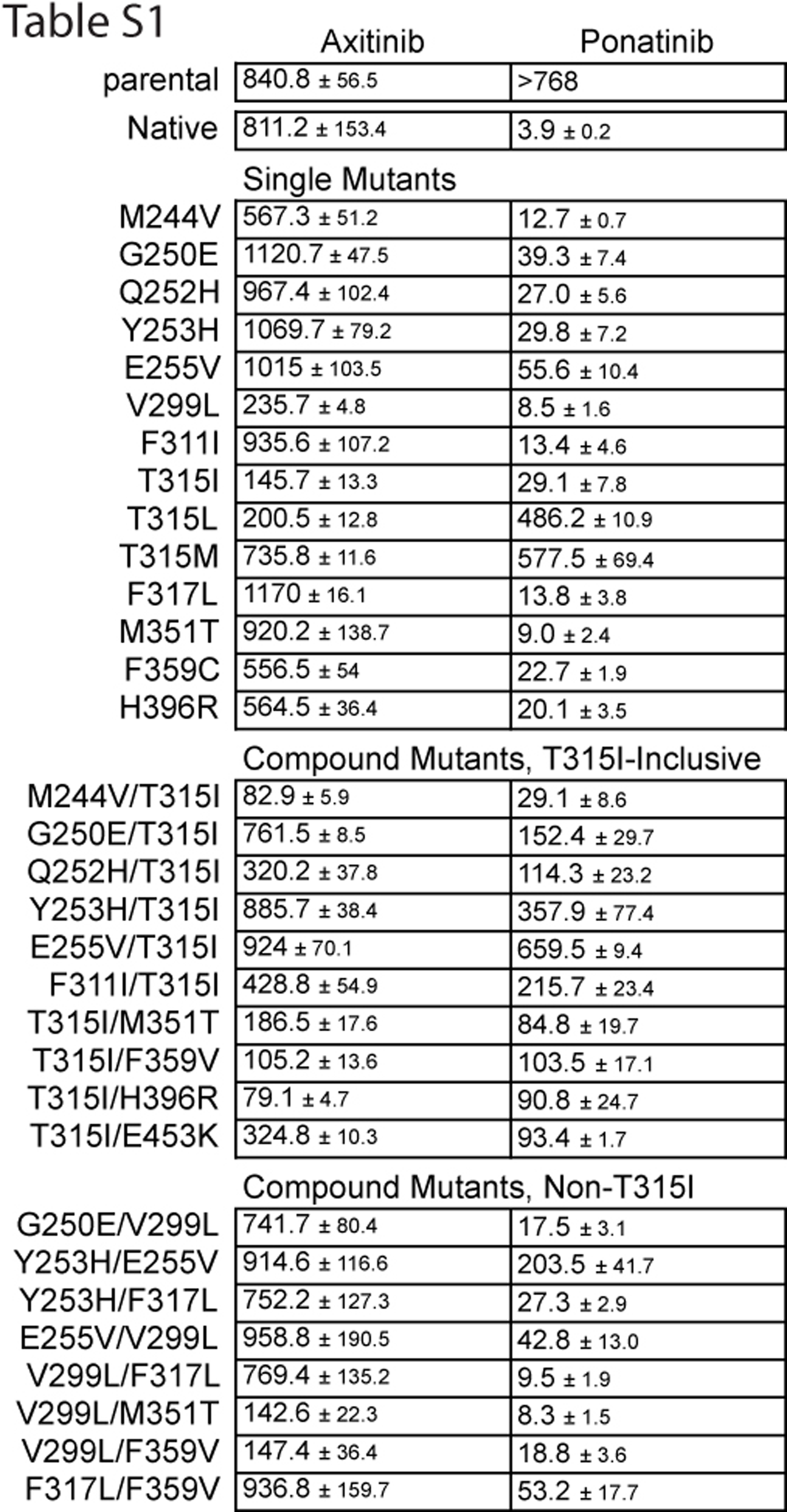

Supplement: Supplementary Table 1 [file leu2015318x3.tif]

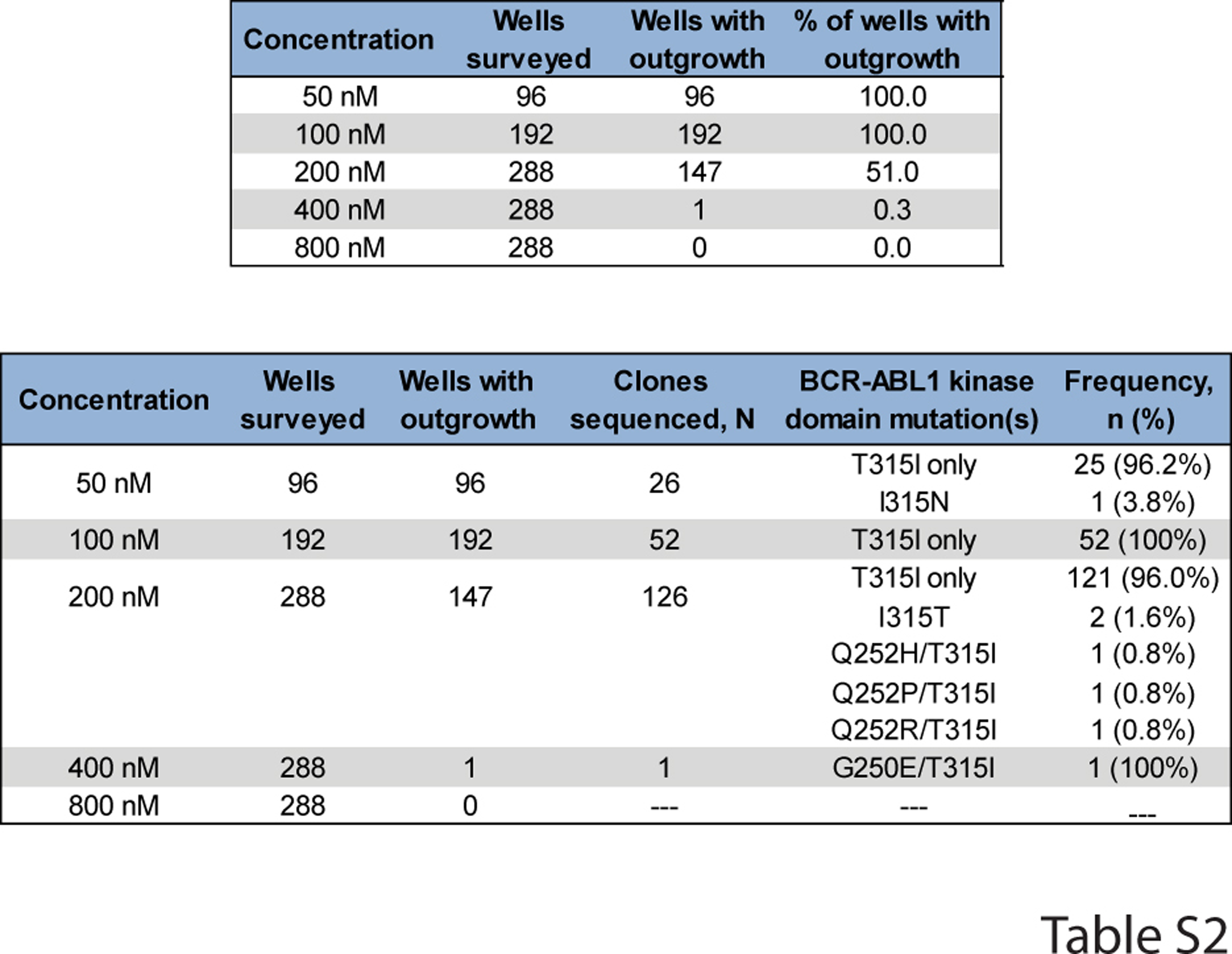

Supplement: Supplementary Table 2 [file leu2015318x4.tif]

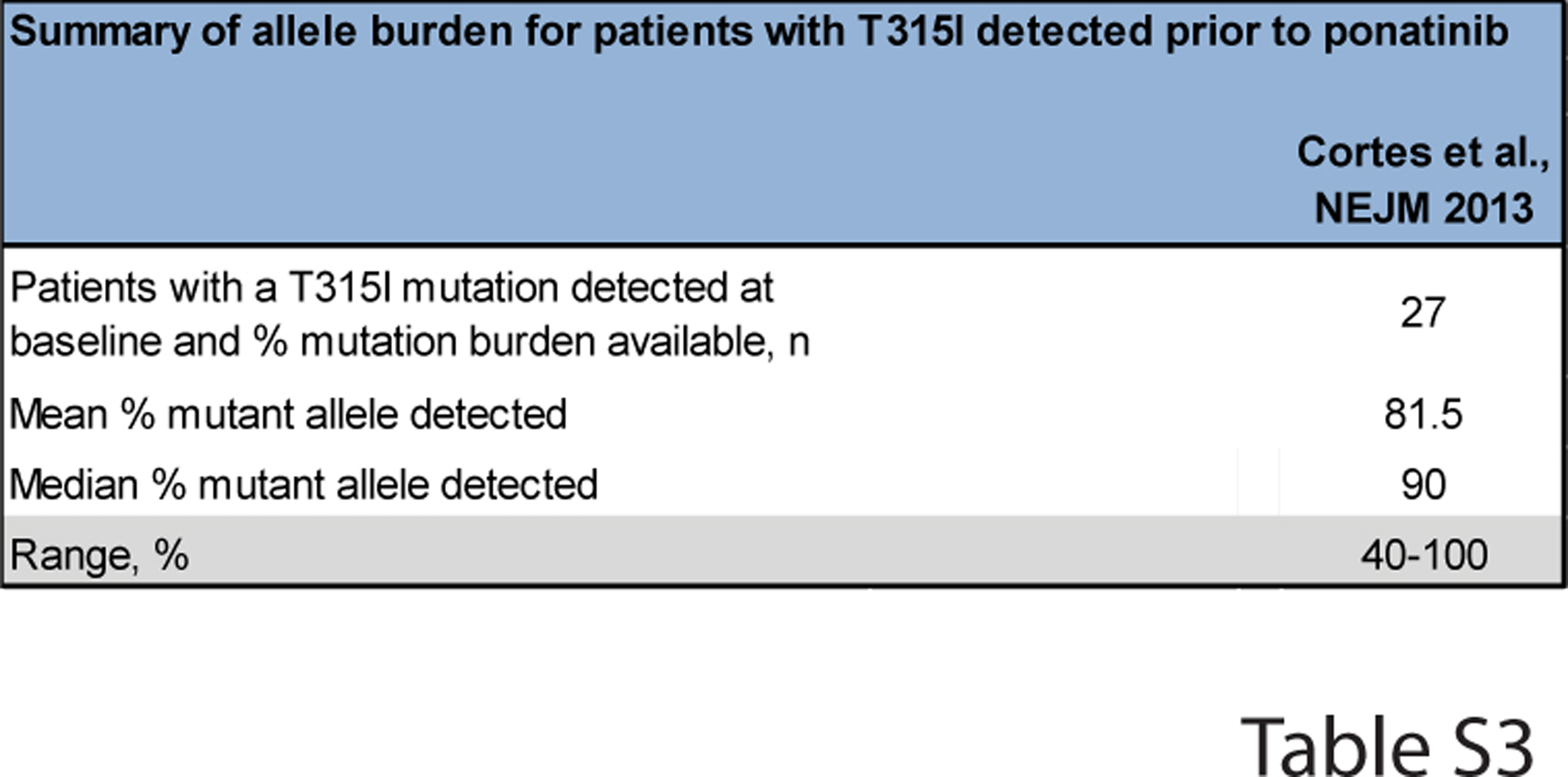

Supplement: Supplementary Table 3 [file leu2015318x5.tif]
