# Supplementary material for: Extreme mutational selectivity of axitinib limits its potential use as a targeted therapeutic for BCR-ABL1-positive leukemia
Source: Leukemia. 2015 Dec 8;30(6):1418–21. doi: 10.1038/leu.2015.318 (PMC4873472; doi:10.1038/leu.2015.318)
Supplement: Supplementary Figure and Table Legends [file leu2015318x6.docx]

**Figure S1. Docking simulations of axitinib identify structural factors that limit tolerated substitutions at position 315 and explain high-level resistance of Y253H/T315I.** Evaluation of the dynamics of ABL kinase mutants (**a**, **b**) ABL^T315I^, (**c**) ABL^T315M^ and (**d**) ABL^Y253H/T315I^ and binding energetics of axitinib using computational molecular dynamics (MD) simulation. Based on docking studies, axitinib occupies the ATP binding pocket in the active state of ABL^T315I^ with favorable interaction energetics (docking score: -14.5 kcal/mol), while displaying a comparatively poor *in silico* affinity towards native ABL, ABL^T315M^ or ABL^Y253H/T315I^ (docking scores: -8.5, -7.5 and -6.4 kcal/mol, respectively). These observations are in agreement with experimental results (see Figure 1). Analysis of docking poses also revealed structural features potentially responsible for the experimentally observed differences in binding affinities. The sulfanyl benzamide group of axitinib showed favorable van der Waals interactions with the aliphatic side chain of T315 in ABL^T315I^ and participated in additional hydrogen bonding with hinge region residues E316 and F317 (**a**, **b**). Native ABL (T315) lacks these hydrophobic interactions, while the T315M mutation introduces a direct steric clash with axitinib binding, in both cases hampering the binding of axitinib (**c**). In the case of ABL^Y253H/T315I^, MD simulation showed that the P-loop undergoes substantial structural rearrangement upon mutation, displacing and partially exposing the H253 residue to solvent, thereby eliminating favorable axitinib interactions with the P-loop (**d**). Specifically, in the context of ABL^T315I^ kinase, Y253 participates in π-π stacking interactions with the indazole ring of axitinib, while ABL^Y253H/T315I^ lacks such interactions. In addition, Y253 participates in hydrogen bonding interactions with the pyridine nitrogen of axitinib. Together, these interactions modulate the binding of axitinib and mutations that change the configurational geometry of these contact positions compromise axitinib binding.

**Figure S2. Axitinib suppresses resistant outgrowth in a cell-based mutagenesis screen and exhibits few BCR-ABL1 compound mutant vulnerabilities**. (**a**) Percentage of wells with outgrowth in graded concentrations of axitinib. (**b**) Resistant subclones recovered from ENU-treated Ba/F3 cells expressing BCR-ABL1^T315I^ cultured with graded concentrations of axitinib (50-800 nM). Each bar represents the percentage of the indicated BCR-ABL1 kinase domain T315I mutant or T315I-inclusive compound mutant among recovered subclones. The percentage of surviving resistant subclones is affected by axitinib. Consequently, a different number of sequenced subclones are represented in the graph for each concentration of axitinib (Supplemental Table S2).

**Table S1. Cell proliferation IC_50_ values of TKIs in parental Ba/F3 cells and Ba/F3 cells expressing native or mutant BCR-ABL1**

**Table S2. Tabulated single-agent axitinib mutagenesis data (starting from Ba/F3 BCR-ABL1^T315I^)**

**Table S3. Summary of allele burden for patients with T315I detected prior to ponatinib**
